# Assessing the efficacy of eDNA metabarcoding for measuring microbial biodiversity within forest ecosystems

**DOI:** 10.1038/s41598-020-80602-9

**Published:** 2021-01-15

**Authors:** Zachary S. Ladin, Barbra Ferrell, Jacob T. Dums, Ryan M. Moore, Delphis F. Levia, W. Gregory Shriver, Vincent D’Amico, Tara L. E. Trammell, João Carlos Setubal, K. Eric Wommack

**Affiliations:** 1 Department of Plant and Soil Sciences, University of Delaware, 264 Townsend Hall, Newark, DE 19716 USA; 2 Department of Plant and Soil Sciences, Delaware Biotechnology Institute, University of Delaware, Newark, DE 19716 USA; 3 Biotechnology Program, North Carolina State University, Raleigh, NC 27695 USA; 4 Department of Entomology and Wildlife Ecology, University of Delaware, 250 Townsend Hall, Newark, DE 19716 USA; 5 Departments of Geography and Spatial Sciences and Plant and Soil Sciences, University of Delaware, 216C Pearson Hall, Newark, DE 19716 USA; 6 US Forest Service, Northern Research Station, Newark, DE USA; 7 Instituto de Química, University of Sao Paulo, São Paulo, SP 05508-000 Brazil

**Keywords:** Biodiversity, Forest ecology, Microbial ecology

## Abstract

We investigated the nascent application and efficacy of sampling and sequencing environmental DNA (eDNA) in terrestrial environments using rainwater that filters through the forest canopy and understory vegetation (i.e., throughfall). We demonstrate the utility and potential of this method for measuring microbial communities and forest biodiversity. We collected pure rainwater (open sky) and throughfall, successfully extracted DNA, and generated over 5000 unique amplicon sequence variants. We found that several taxa including *Mycoplasma* sp., *Spirosoma* sp., *Roseomonas* sp., and *Lactococcus* sp. were present only in throughfall samples. *Spiroplasma* sp., *Methylobacterium* sp., *Massilia* sp., *Pantoea* sp., and *Sphingomonas* sp. were found in both types of samples, but more abundantly in throughfall than in rainwater. Throughfall samples contained Gammaproteobacteria that have been previously found to be plant-associated, and may contribute to important functional roles. We illustrate how this novel method can be used for measuring microbial biodiversity in forest ecosystems, foreshadowing the utility for quantifying both prokaryotic and eukaryotic lifeforms. Leveraging these methods will enhance our ability to detect extant species, describe new species, and improve our overall understanding of ecological community dynamics in forest ecosystems.

## Introduction

Forest ecosystems are highly complex and dynamic assemblages of biotic and abiotic networks that support diverse biological communities^1^. Forests are also critical to numerous ecological functions from global to local scales^2^, which include mediating water cycling, nutrient cycling, carbon storage, and providing habitat for billions of species across the planet^3^. Continued increasing rates of globalization and concomitant anthropogenic effects are leading drivers of forest habitat loss, threatening species diversity^4,5^. Moreover, exacerbating factors such as climate change and biological invasion additionally reduce habitat quality, which may lead to disproportionate losses of biodiversity related to contemporary losses of forest habitat, globally^6^. For these reasons, it is imperative that we gain a better understanding and more accurate means of quantification of the ecological communities that comprise these ever-threatened ecosystems.

The long-standing importance of understanding ecosystems and ecological communities has largely focused on the dynamics of synergistic interactions among organisms and abiotic components^7–9^. Despite the prevalence of theories and differences in approaches to finding underlying generalizable rules^10^ for describing and modeling community-level patterns^11,12^, understanding ecological communities requires the accurate measurement of biological diversity^13^ hereafter ‘biodiversity’ as pertaining to organismal or species diversity^14^. Leveraging rapid technological advances in recent decades, such as high-throughput sequencing, we are now able to characterize community assemblages within ecosystems with increasing accuracy and detail^15^. Furthermore, unprecedented perspectives of biodiversity and complexity within communities has also provided new frameworks for approaching the nature of interrelations that can influence community dynamics^16,17^. Within forest ecosystems in particular, the elucidation of interactions among plant-soil-microbiome associations^18^ can provide new insight in understanding forest health and function more generally^19,20^.

Over the past decade, environmental DNA (eDNA) has become an important method of detecting species occurrence^21–24^. Here, we follow the definition of eDNA as a “complex mixture of genomic DNA from many different organisms found in an environmental sample^25^.” Sampling and analyzing eDNA is made possible using high-throughput sequencing and metabarcoding methods that are revolutionizing how we can now approach inventory and monitoring of biological diversity^26–29^. For example, eDNA metabarcoding has been effectively used to detect presence of plants^30^, fungi^31^, animals^32,33^, and microorganisms^34^. Use of eDNA metabarcoding has been especially well-suited for identifying cryptic, rare, or endangered species. For example, eDNA from water samples has been successfully used to identify the presence of a rare and endangered saw-toothed fish^35^, and similarly, eDNA has been used to detect the presence of an endangered marine skate species^36^.

In general, using eDNA metabarcoding has many practical applications that include detecting biological invasion^37–39^, trophic ecology^40,41^, reconstructing ancient ecosystems^42^, plant-pollinator interactions^43,44^, and monitoring air and water quality^45–47^. Despite the impressive use of eDNA metabarcoding as a tool to increase our ability to measure biodiversity, eDNA metabarcoding has been limited to applications which use aquatic samples containing DNA, given the inherently fragile nature of organismal tissue containing DNA and its rapid degradation when exposed to typical environmental forces^48^. For instance, several studies have explored degradation processes of eDNA within aquatic environments^49,50^. Another study exploring persistence and factors influencing eDNA within soils found that eDNA became undetectable after roughly one week, although this may vary as a function of soil moisture, temperature, and habitat characteristics^51^. However, despite these methodological challenges, innovative use of eDNA metabarcoding is increasingly being used to detect species within terrestrial ecosystems^52–55^.

It has also been well established that airborne biological particles occur within the atmosphere^56^, and several studies have been successful in sampling and sequencing eDNA from airborne samples^46,57–59^. However, very few studies to date have explored the partitioning of microbial communities contained and transported in pure rainwater and throughfall^60,61^. Throughfall is the incident precipitation that passes either directly through the forest canopy, or alternatively is first intercepted and splashed from the canopy or coalesces and drips down to lower strata^62,63^. Along with stemflow, which is the intercepted precipitation that flows along branches and converges on the stem of plants, this canopy-breaching throughfall input can constitute upwards of 90% of precipitation entering a forest^64^. Key to hydrologic and biogeochemical cycling within forests, throughfall and stemflow transport water, solutes, particulates, and nutrients to the soil, where inputs enrich soils thereby influencing growth and dynamics of vegetation^65–67^ and crops^68^.

Airborne microorganisms that are found in aerosols can also play a role in cloud formation via ice nucleation creating biogeochemical feedback loops with vegetated areas, such as forests, indicated by the bioprecipitation hypothesis^69^. Indeed, studies that have successfully sequenced eDNA within aerosols^59^ also lend further support to the bioprecipitation hypothesis^70^. Our study was specifically motivated to further assess the efficacy of not only sequencing eDNA within rainwater, which holds interesting implications unto itself, but to see if we could sequence eDNA from organisms that come into physical contact with rainwater in the forest canopy and understory, enabling rain droplets to acquire and sequester eDNA for sampling. By sampling rainwater that filtered through the forest canopy and understory vegetation we were able to successfully obtain DNA and quantify microbial consortia within terrestrial forest ecosystems. We predicted that throughfall would contain greater taxa diversity and greater bacteria abundances than rainwater that did not contact forest vegetation. We wanted to test the hypothesis that there would be additive effects of bioprecipitation and collection of forest canopy phytobiome communities compared to rainwater alone. This is important given the demonstrated linkages of and need for plant-associated bacteria and key functional roles these interactions can have (e.g., nitrogen fixing within the phylloshphere^61,71^.

## Materials and methods

### Study area

**Figure 1:**
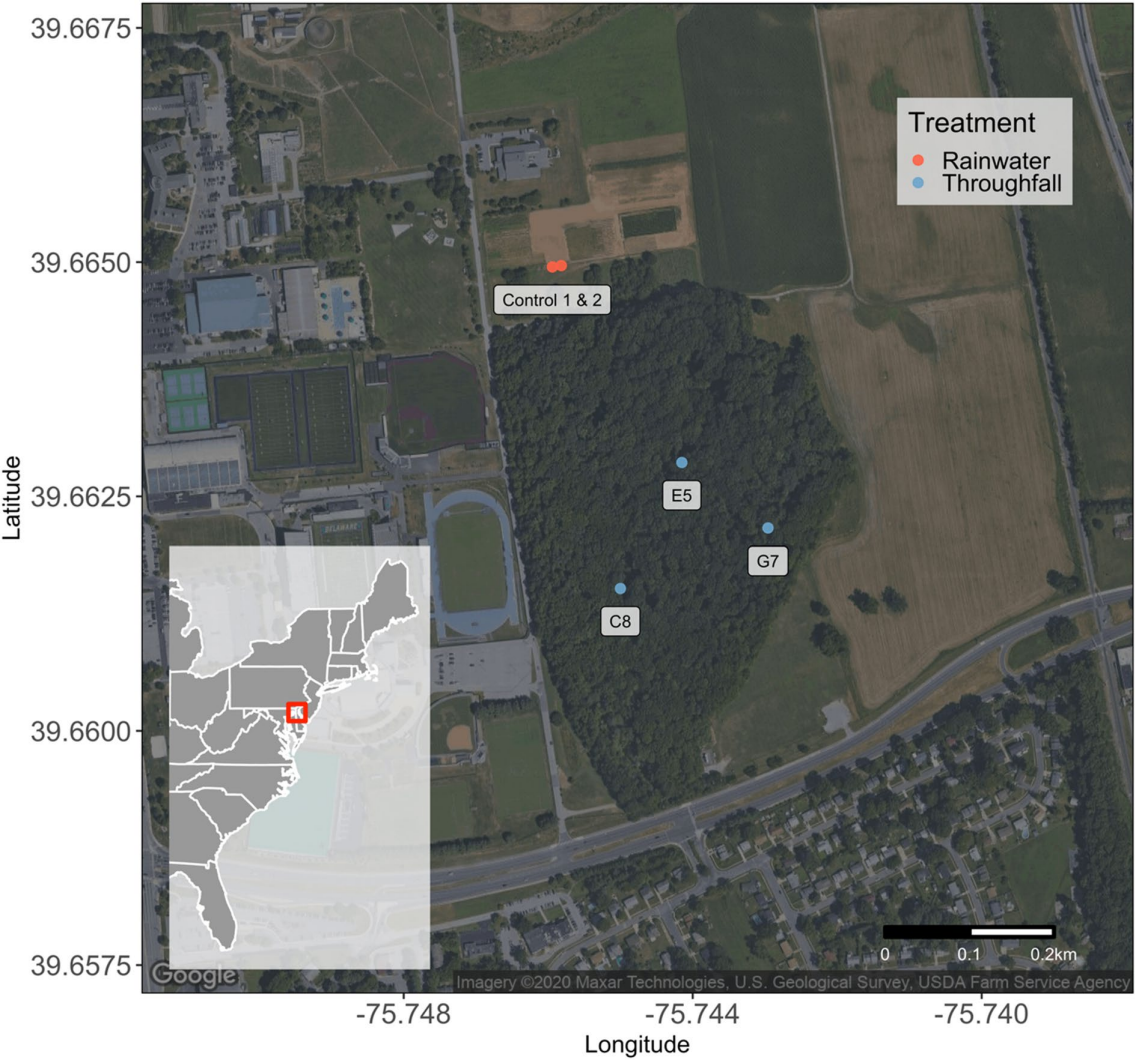
Map of study area showing sampling locations of rainwater (red) and throughfall (blue) treatments within and adjacent to the 16-ha forest (− 75.74477 W, 39.66249 N) in Newark, Delaware, USA. Map produced using program R (ver. 4.0.2; https://www.r-project.org/).

Our study occurred within and near a 16-ha forest located in Newark, Delaware, USA (− 75.74477 W, 39.66249 N) within the mid-Atlantic United States (Fig. 1). This small forest consists of a vegetation community with dominant canopy tree species including *Fagus grandifolia* Ehrh (American beech), *Acer rubrum* L. (red maple), *Quercus* spp*.* (oak), *Liriodendron tulipifera* L. (tulip poplar), and *Liquidambar styraciflua* L. (sweetgum). Additionally, this forest contains both native and non-native understory woody species including *Lindera benzoin* L. (spicebush), *Viburnum* spp. L. (arrowwood viburnum), *Clethra alnifolia* L. (sweet pepperbush), *Rosa multiflora* Thunb. (multiflora rose), *Eleagnus umbellate* Thunb. (autumn olive), and *Rubus* spp (brambles). We randomly selected three sampling points from a suite of previously-established gridded points^72^ each of which were greater than 50 m apart to achieve spatial independence, and 50 m from the forest edge to reduce bias from potential edge effects. We also located two rainwater sampling locations north of the patch on the edge of an adjacent fallow agricultural field that was over 100 m from the forest edge.

### Sample collection

We collected samples of rainwater (*N* = 3) and throughfall (*N* = 10) in Newark, Delaware, USA from a single rain event on 11 August 2018. We collected samples at 3 randomly-selected locations within a 16-ha forest, and 2 open locations north of the forest within an adjacent field (Fig. 1). On 10 August 2018, one hour before an anticipated rain event between 1730 and 1800 h, we deployed two replicate high-density polyethylene 45.72-cm diameter funnels (United States Plastic Corp, Lima, Ohio, USA; see Fig. S1 in Supplementary Materials) at each sampling location spaced roughly 5 m apart. Each funnel was surface sterilized within the laboratory using 100% EtOH, dried, and stored within sterile plastic bags until deployment. Additionally, each funnel was fitted with a sterilized 10.16-cm diameter funnel screen (0.5 mm mesh) to keep large debris from falling into rainwater samples. Each funnel was attached to a straight metal post using plastic zip-ties at a ca. 1-m height above the ground. Upon deployment, each funnel was rinsed with 50 mL of sterile de-ionized water, and these samples (*N* = 8), were used as an internal control sample to detect any background bacterial DNA contamination in the sampling apparatus. These ddH2O samples were immediately brought to the laboratory, flash frozen in liquid nitrogen, and stored frozen at -80 °C for DNA analysis to detect any potential contamination. Finally, we blocked the bottom of the funnels using sterilized No. 7 rubber stoppers (Fisher Scientific Company, Waltham, Massachusetts, USA). All water samples were collected within 50-mL conical sterile polypropylene centrifuge tubes (Fisher Scientific Company, Waltham, Massachusetts, USA). We then returned the following day (11 August 2018) at 0700 h to collect rainwater samples, which were then immediately transferred to the laboratory, flash frozen in liquid nitrogen, and stored frozen at -80 °C until analysis^73^.

### Sample processing and 16S sequencing

Frozen samples were removed from -80 °C and immediately thawed in a warm water bath (50 °C), and then passed through sterile 0.22-µm Sterivex filter units without filling bell (Cat# SVGP010, Millipore, Burlington, Massachusetts, USA) using sterile 10-mL B-D Luer-Lok Tip syringes (Fisher Scientific Company, Waltham, Massachusetts, USA). We then extracted DNA from each of the filters using Qiagen DNeasy PowerWater kits (Qiagen, Hilden, Germany). DNA concentration was assessed using a Qubit 4 Fluorometer (Fisher Scientific Company, Waltham, Massachusetts, USA). A total of 21 samples (5 sampling locations each with 2 replicates, 10 ddH2O control samples, 1 DNA extraction control and 1 PCR no template control) were used to generate 16S rRNA amplicon libraries. The V4-V5 region of the 16S rRNA gene was amplified from 12.5 ng of DNA using the primers (515F-Y (5′-GTGYCAGCMGCCGCGGTAA) and 926R (5′-CCGYCAATTYMTTTRAGTTT)^74^ with the Illumina sequencing adapters attached. We chose to use these standard primers which have been previously undergone in silico and in vitro validation^74^ to allow comparison of our results to previous research. Moreover, these primers were developed to cover an expanded range of organisms wide enough to capture additional eukaryotic sequences as well^74,75^. Each sample was barcoded following Illumina MiSeq standard procedures for Nextera indices. Samples were sequenced using paired-end 2 × 360 bp on the Illumina MiSeq as part of a 183 total sample equimolar pool at the University of Delaware—Sequencing and Genotyping Center.

### Analysis of sequence data

Sequence data were analyzed by first removing forward (515F-Y) and reverse (926R) 16S primers and merging the demultiplexed reads with FLASH (ver. 1.2.11)^76^, which were then pooled. We then used the fastp function (ver. 0.20.0)^76^ to remove any reads with > 40% of bases having a quality score of < 20, remove any reads having more than one ambiguous base, and remove bases from both the front and back of a given read, when a 4-base sliding window average quality score was < 25. After confirming that primers were successfully removed, mean quality scores for reads ranged between 24 and 38. We then dereplicated our reads using VSEARCH (version 2.13.4)^78^ to identify, merge, and sort identical sequences by decreasing abundance. Subsequently, we generated amplicon sequence variants (ASVs) using the unoise3 algorithm^79^ implemented via VSEARCH (version 2.13.4)^78^. We then checked for and removed potential chimeras within reads using the ‘–uchime_denovo’ function within VSEARCH (version 2.13.4)^78^. We removed all ASVs that were uniquely detected in ddH2O samples prior to analyses of rainwater and throughfall samples, and report these omitted ASVs (see Table S2 Supplementary Materials). All analyses were implemented with program R (ver. 4.0)^80^.

### Taxonomic assignment

Due to potential contamination during sequencing, we did not include the ddH2O sample from the Control 2 sampling location within any further analyses. We used the ‘vsearch–sintax’ function, a non-Bayesian classifier that uses a *k*-mer matching and bootstrapped confidence interval estimation algorithm for assigning taxonomies to ASVs^81^. However, given the potential for incorrect assignment^82,83^, we performed all analyses on ASVs directly, and subsequently for exploratory purposes, presented taxonomic assignment of data up to the rank of genus.

### Statistical analysis

After examining the number of reads for each ASV, we discarded any ASVs with reads < 5 (i.e., a 0.1% minimum abundance filter) within our data to avoid inclusion of sequencing errors^84^. After pooling within-sample replicates (i.e., in cases where more than one cryovial of rainwater was collected at a sampling point), we excluded any samples with < 2,000 reads. This list of unique ASVs found within ddH2O samples were then removed from the data, and not included in further analyses. We grouped data categorically into unique ASVs found only within throughfall samples, ASVs found in both throughfall and rainwater samples, and ASVs found only within rainwater samples, and then used a nonparametric Kruskal–Wallis rank sum test to determine significant differences among groups followed by test of multiple comparisons^85^ to determine pair-wise differences in counts of ASVs between groups.

Due to the compositional nature of these data^86,87^, we first transformed counts of ASVs per sample to the relative proportions, and then to centered log-ratios^88^ using the acomp() and clr() functions within the ‘compositions’ (ver. 1.40–5) R package^89^, respectively. Given the sparse nature of our data having a high proportion of zeros within our counts, we used a square-root Bayesian-multiplicative replacement of zeros^90^ with the cmultRepl() function from the ‘zCompositions’ R package (ver. 1.3.4)^91^.

To assess differences in community composition and structure between throughfall and rainwater, we independently computed Aitchison distances, which are the pair-wise Euclidean distances of centered log-ratio transformed ASV data^88^, among sampling locations, plots, and between treatments using the dist() function within the ‘compositions’ package (ver 1.40–5)^89^. We subsequently used a nonparametric Kruskal–Wallis rank sum test to test for differences between throughfall and rainwater samples using plot-level Aitchison distances as the response variable.

We also estimated alpha diversity using the package ‘DivNet’ (ver 0.3.6)^92^ to test for differences in microbial community diversity (at the genus rank) between throughfall and rainwater and among sampling plots. We estimated alpha diversity and beta diversity non-directional variation in community structure^93^ following suggested methods that use appropriate log-transformed ratios of count data assuming communities are interconnected networks and hence, compositional in nature when estimating alpha and beta diversity^92,94–96^. Alpha diversity (Shannon’s diversity index) and beta diversity (Bray–Curtis dissimilarity) indices were estimated for throughfall and rainwater were computed using the ‘breakaway’ package (ver. 4.7.1)^97^. We finally visually assessed patterns in community composition between throughfall and rainwater treatments by looking at mean and coefficient of variation (CV) of counts among genera and visualizing a hierarchical taxonomic tree. All statistical analyses were performed using program R (ver. 4.0.0)^80^.

## Results

We successfully extracted DNA from collected ddH2O water, rainwater, and throughfall samples. Sample volumes ranged between 1 and 40 mL, and DNA concentrations within samples ranged between trace amounts < 0.050 to 2.5 ng per µL. All samples with relatively low DNA trace concentrations were either from ddH2O or from rainwater (Table S1 in Supplementary Materials). After first pooling ASV data from sub-samples (i.e., samples from C8_1, E5_1, E5_2, G7_1, G7_2, where we collected and independently analyzed two samples per funnel), and removing all samples with fewer than 2,000 reads, the number of reads per sample ranged between 4922 and 196,015 with a mean of 93,200 (see Fig. S2 in Supplementary Materials). Within each treatment (i.e., throughfall and rainwater) the mean number of reads for throughfall and rainwater samples were 68,896 and 32,960, respectively.

**Figure 2:**
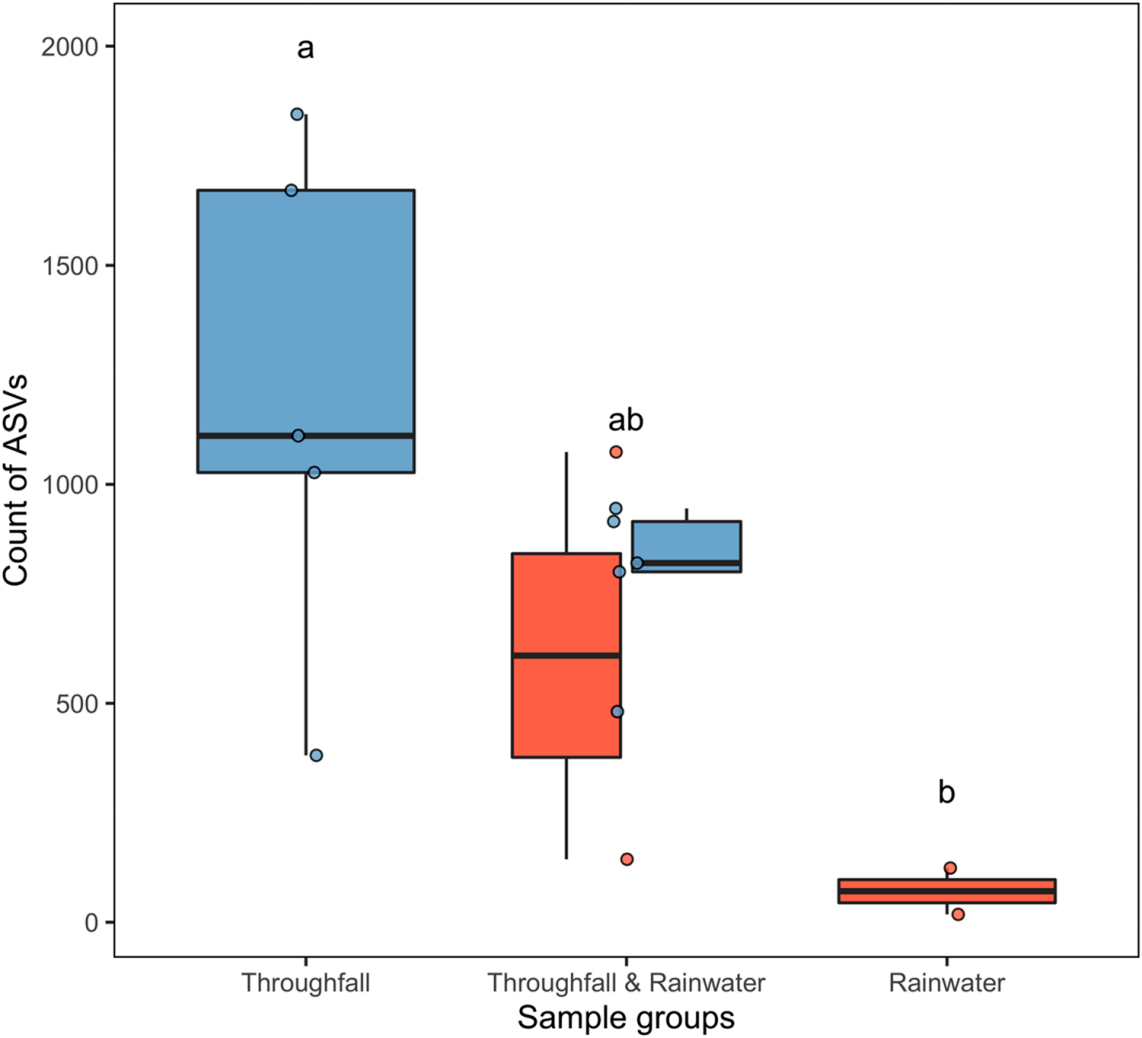
Box-and-whisker plots showing the difference in the count of unique ASVs detected among sampling groups: throughfall, throughfall and rainwater, and rainwater. Rainwater and throughfall treatments are shown in red and blue, respectively. Black lines denote medians, boxes represent 75% quantiles, red and blue dots show sample values, and lowercase letters indicate significant pair-wise differences (α = 0.05).

On average among samples, there were 4,065 unique ASVs ranging between 352 and 3614 ASVs (Supplementary Materials). Throughfall and rainwater samples had mean counts of 3,066 and 1,443 ASVs, respectively. The number of unique ASVs (mean ± SE) differed among groups (Kruskal–Wallis chi-sq. = 6.82, df = 2, *P* < 0.05), and the count of ASVs found solely within throughfall samples (1170.4 ± 266) was greater than those found in rainwater-only samples (81 ± 58; Z = − 2.51, *P* < 0.05, Fig. 2). However, the mean number of ASVs found in both throughfall and rainwater samples were similar to both ASVs found either in throughfall-only (Z = − 1.67, *P* = 0.14; Fig. 2) or rainwater-only groups (Z = − 1.40, *P* = 0.16; Fig. 2).

**Figure 3:**
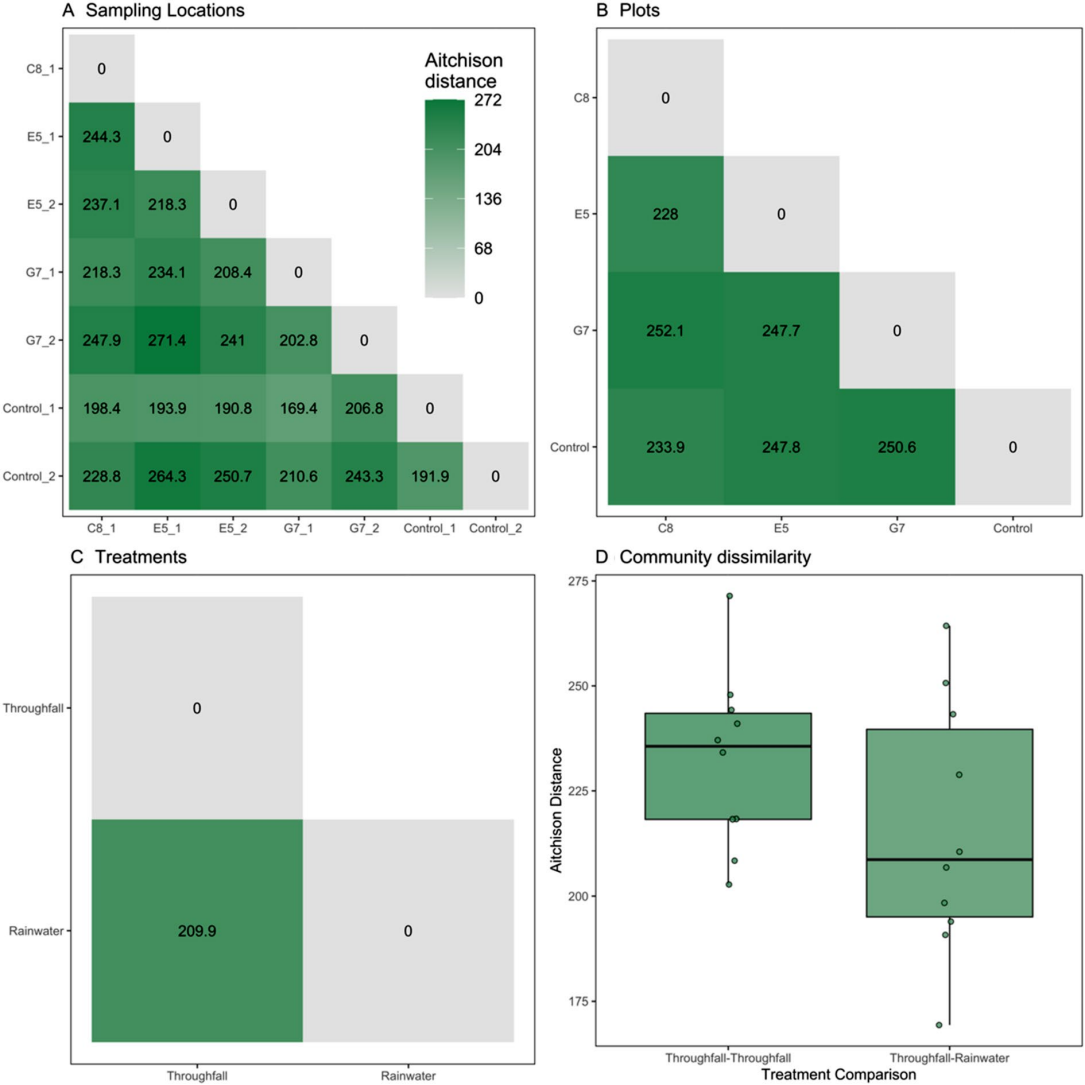
Plots of community dissimilarity based on pair-wise Aitchison distances (i.e., centered log-ratio transformed relative proportions of ASVs) among (**A**) sampling locations, (**B**) plots, and (**C**) throughfall and rainwater treatments. Aitchison distances are shown by a color ramp from light gray to green. A boxplot-and-whisker plot (**D**) compares plot-level mean Aitchison distances within throughfall samples, and between throughfall and rainwater treatments.

The degree of community dissimilarity inferred here based on Aitchison distances among all sampling locations (including both throughfall and rainwater treatments) ranged between 169.4 and 271.4, where 0 indicates having no dissimilarity, and greater values represent increasing dissimilarity (Fig. 3A). Pair-wise Aitchison distances among four plots ranged between 228.0 and 252.1 (Fig. 3B). The Aitchison distance between throughfall and rainwater treatments was 209.9 (Fig. 3C). When comparing the variation of within- and between-treatment community dissimilarity, we found that mean Aitchison distances were similar between throughfall-throughfall versus throughfall-rainwater treatment comparisons (Kruskal–Wallis chi-sq. = 3.628, df = 2, *P* = 0.16; Fig. 3D).

**Figure 4:**
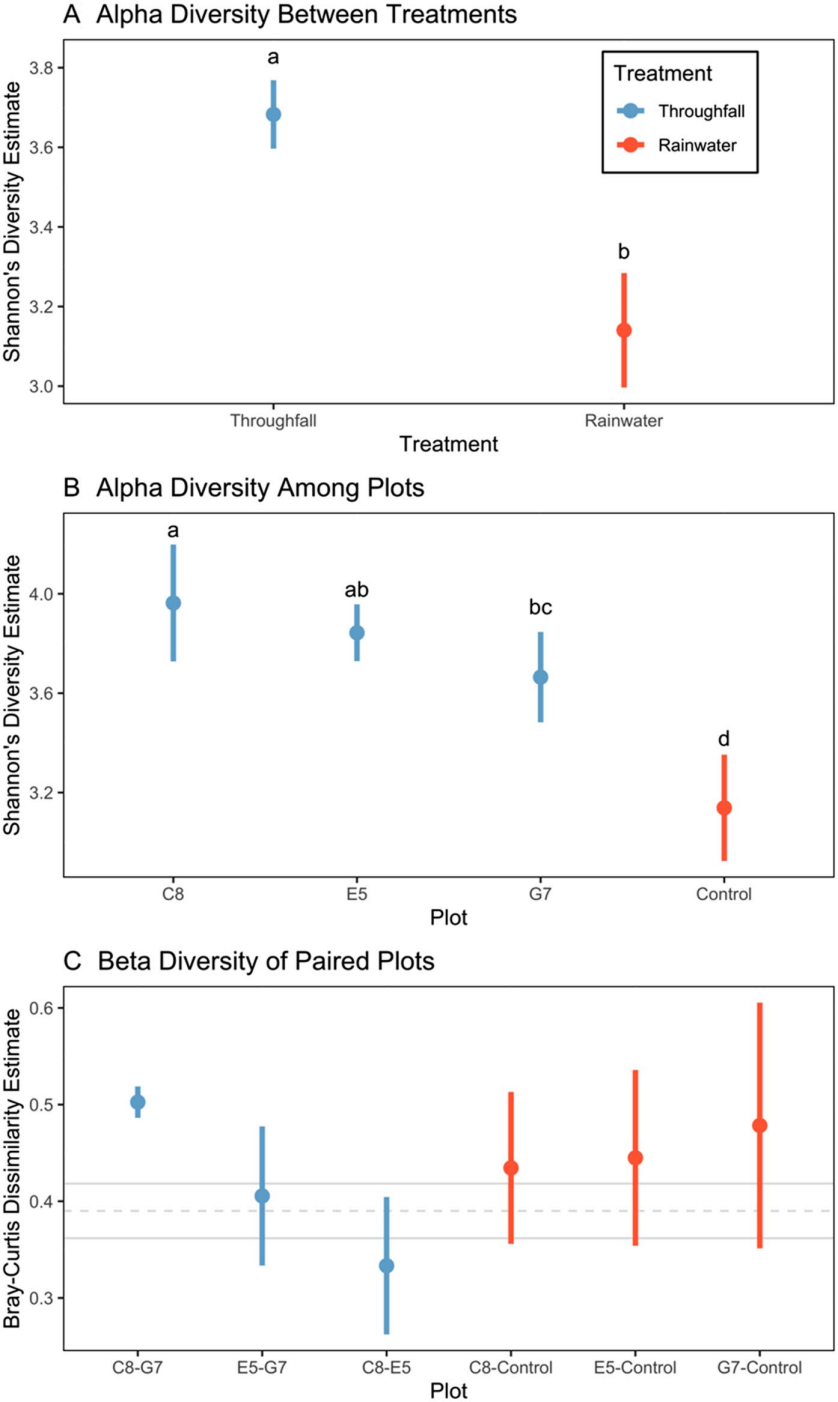
Community diversity indices (mean ± SE) for (**A**) Shannon’s diversity index showing comparison of alpha diversity between throughfall (blue) and rainwater (red) treatments, (**B**) alpha diversity among plots, and (**C**) Bray–Curtis dissimilarity between all unique pairs of plots, with light gray lines indicating mean and SE of community dissimilarity between throughfall and rainwater. Lowercase letters indicate significant differences.

Bacterial communities found in throughfall had a significantly greater alpha diversity (3.68 ± 0.086), as indicated by Shannon’s diversity estimate (mean ± SE), than rainwater (3.14 ± 0.14; *Q*-statistic = 10,125, *P* < 0.0001; Fig. 4A). Alpha diversity differed among plots (*Q*-statistic = 3,785, *P* < 0.0001). Each of the forest plots where throughfall samples were collected had greater alpha diversity, i.e., C8 (3.96 ± 0.16), G7 (3.65 ± 0.25), and E5 (3.65 ± 0.25) compared to control (rainwater) plot (3.14 ± 0.302; *ß* = − 0.299, P < 0.05; Fig. 4B). Alpha diversity was greater at C8 compared to G7 (*ß* = − 0.2986, *P* < 0.05). However, pair-wise hypothesis tests indicated that alpha diversity was similar between C8 and E5 (*ß* = − 0.1198, *P* = 0.14), and between E5 and G7 *ß* = − 0.1718, *P* = 0.13; Fig. 4B). Bray–Curtis dissimilarity estimate (mean ± SE) between throughfall and rainwater treatments was 0.39 ± 0.03. However, we did not detect a difference in Bray–Curtis dissimilarity estimates among any of the independent comparisons between plot pairs (*P* > 0.17 in all cases; Fig. 4C).

**Figure 5:**
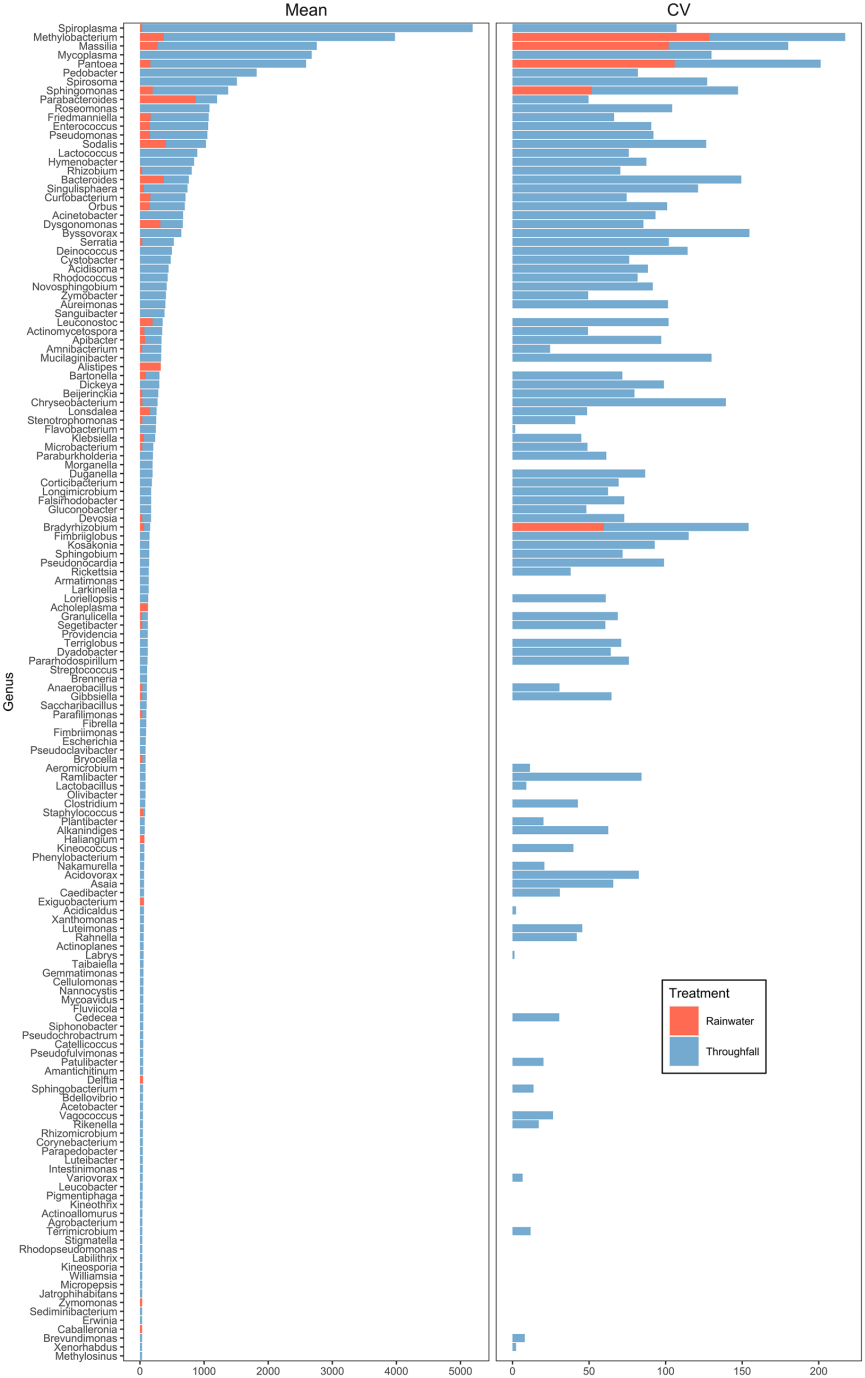
Mean abundance (number of ASV reads) and coefficient of variation (CV) for taxonomically-assigned ASVs for 151 unique bacterial genera detected in rainwater (red) and throughfall (blue) samples.

**Figure 6:**
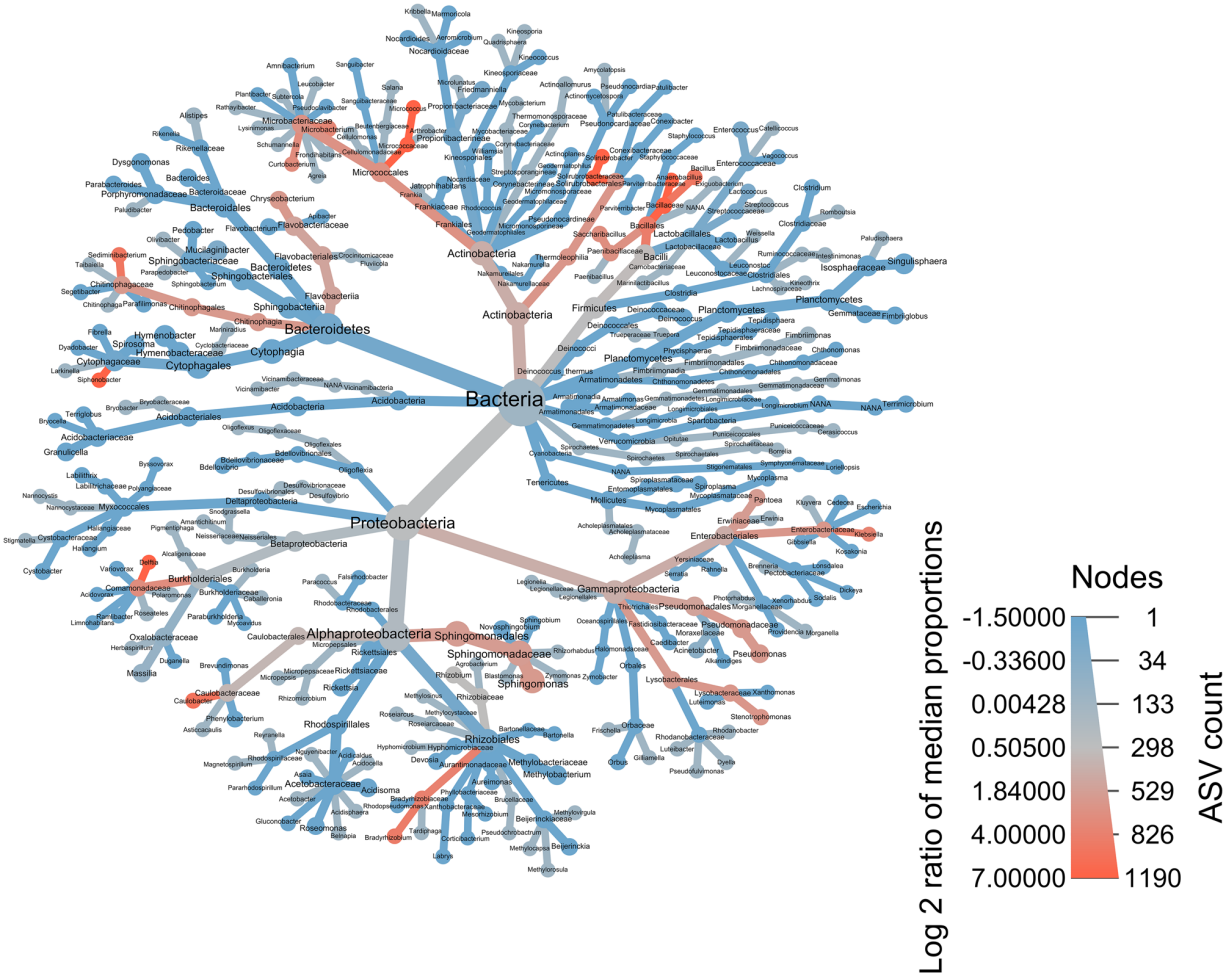
Taxonomic tree showing hierarchical structure of taxonomic ranks (denoted at each graph node) of 221 unique genera, and their membership in community assemblages based on the log_2_ of median ratios of genera detected within throughfall (blue), rainwater (red) and taxa found in both throughfall and rainwater (gray) samples. Thickness of graph edges indicate relative abundance of taxa. This figure was produce using the ‘metacoder’ package (ver. 0.3.4; https://grunwaldlab.github.io/metacoder_documentation/index.html)^77^ in program R (ver. 4.0.2).

In general, the abundance (mean and CV) differed among taxonomically-assigned genera (Kruskal–Wallis chi-sq. = 270.6, df = 149, *P* < 0.0001; Fig. 4). Genera assigned to ASVs that were found in both throughfall and rainwater samples were in general found in greater abundance in throughfall, including *Spiroplasma, Methylobacterium*, *Massilia*, *Pantoea*, and *Sphingomonas* (Fig. 5). Genera found only in throughfall samples include *Mycoplasma*, *Pedobacter*, *Spirosoma*, *Roseomonas*, and *Lactococcus*. (Fig. 5). Among the top 20 in abundance, only ASVs associated with *Parabacteroides* were more abundant in rainwater samples than in throughfall (Fig. 5). Rainwater-only samples were lower in richness (the number of unique taxa), and in general were found in lower abundances, and included genera such as *Parabacteroides* sp., *Kluyvera* sp., and *Xanthomonas* sp. (Fig. 5). Further, visual examination of a hierarchical tree of taxonomically-assigned lineages to ASVs using the ‘metacoder’ (ver. 0.3.4) R package^98^ also showed that throughfall contained greater diversity than rainwater (Fig. 6).

## Discussion

We demonstrated the successful sampling and sequencing of eDNA contained within throughfall and rainwater, and assessed community composition patterns using an appropriate compositionally-aware framework^86,92,95^. This opportunistic method is capable of measuring microbial biodiversity uniquely associated with terrestrial forest ecosystems. We predicted that throughfall-derived samples would contain greater taxa richness and that these taxa would, in general, would be found in greater abundances, both of which we found to occur (Figs. 4, 5). Our findings complement and lend further support for the promise of using eDNA metabarcoding as a tool for measuring and monitoring biodiversity^99–102^.

While our prediction that throughfall samples would contain more diverse taxa than rainwater samples was supported, we also were interested in investigating how diversity might differ between throughfall and rainwater treatments (alpha diversity), among sampling locations (beta diversity), and the amount of within-treatment variation. We discovered that there was a high degree of community dissimilarity (interpreted as ecological distance) both within- and among-sampling locations as measured via Aitchison distances that account for the compositional nature of microbial community data^86^. For example, communities at sampling location E5 within our study area were more dissimilar than throughfall and rainwater sampling plots (Fig. 3B). Yet, we also found greater dissimilarity within-sampling location replicates at E5 (i.e., E5_1 and E5_2; Fig. 3A). However, when comparing the degree of within-treatment community dissimilarity, we found throughfall samples to have more similar microbial communities than rainwater samples. This observed outcome could result from small sampling efforts that may introduce bias.

Detected differences in alpha diversity (i.e., Shannon’s diversity estimates) between throughfall and rainwater treatments, and associated plot-level comparisons additionally supported our predictions that throughfall would contain greater community diversity. Estimated beta diversity from Bray–Curtis dissimilarity of 0.38 also suggests that communities in throughfall and rainwater were distinct relative to the global taxonomic pool among our samples. Our lack in finding significant differences in beta-diversity among paired plot comparisons is likely due to low sample sizes, which future studies may alleviate with increased sampling. We also understand that many factors can contribute to variation in microbial communities related both to environmental and sampling bias. While our study demonstrates variation within and among treatments, future studies with experimental designs having increased numbers of sampling locations among all treatments, additional within-sampling location replicates, and repeated sampling through time would provide data better-suited to evaluating how local-scale environmental effects (e.g., variation in weather events, vegetation structure, distance-to-edge, wind direction, etc.) may influence microbial diversity in throughfall.

There remain several unknown yet important questions about how microbial communities form and persist within forest ecosystems, how community dynamics over time are influenced by bioprecipitation, and how these might influence forest ecosystem health and function. Quantifying the degree to which bioprecipitation may influence nutrient and mediation of biogeochemical cycling for instance can help better understand how this phenomenon influences forest ecosystems. Supportive results from other studies using flow cytometry to measure bacterial concentrations (cells mL^-1^) found 5.5 times greater concentrations of bacteria in throughfall, and 10 times greater concentrations in stemflow compared to pure rainwater (0.52 × 10^5^ cells mL^-1^)^103^. In our study, we also found greater abundances of ASVs within throughfall samples compared to pure rainwater, supporting our prediction. Although ASV abundance is not analogous to cell concentration per se, our DNA sample concentrations also reflected this pattern (see Table S1 in Supplementary Materials).

It has long been known that microbial communities associated with the phyllosphere and rhizosphere play important roles in biogeochemical cycling of nutrients, and hence forest ecosystem function and dynamics^18,19^. Given the evidence supporting the bioprecipitation hypothesis^103–105^, and the likelihood of microbial agents being transported and input into forests via throughfall and stemflow, more research into this area is warranted. For example, using a combination of measuring δ^17^O and δ^18^O stable isotopes in forests to estimate nitrogen deposition in pure rainwater and throughfall, along with analyzing DNA obtained from leaf surfaces, rainfall, and throughfall, another study found that nitrate (${\mathrm{NO}}_{3}^{-}$) was enriched within throughfall compared to pure rainwater via increased nitrifying microbial activity^61^. Despite limitations in interpreting results from previous studies that have not explicitly accounted for the compositional nature of microbial community data^60,103^ which can introduce bias, our results of relative patterns of taxa found in throughfall and rainwater support previous findings. In a previous study, microbial communities also differed between rainwater and throughfall samples, and we detected 14 of 15 (93%) of the same bacterial phyla (Fig. 6) within our samples that as those detected within their study^61^. Despite not being able to make direct comparisons about community diversity per se*,* in general, we detected 82% of the same classes found in another study that explored how variability in storm events can influence microbial community inputs into forests as well as increasing the transfer of bacteria from the canopy to the forest floor via throughfall and stemflow^60^.

The implications for future explorations into these phenomena are tantalizing, indeed. Take for example the global-scale transport of nearly 40 million tons of dust particulates per year originating in the Bodel´e depression in the Sahara Desert in Africa and ending up in North and South America^106^. This phenomenon, driven by multiple factors including topography, seasonal patterns in global weather and prevailing winds, all of which are influenced by climate change, are known to transport significant amounts of mineral and trace nutrient inputs to the Amazon rainforest in South America^107,108^, and in general, hold important implications for nutrient inputs into forests^109^. Given the overwhelming support of the bioprecipitation hypothesis^70,110–112^, it is likely that sampling Saharan dust, for example, using the eDNA metabarcoding approach set forth here, would yield a treasure trove of microbial community data, and would build upon findings from previous studies^101,113^ to provide further insight into global-sale macroecological dynamics of interconnected yet geographically disparate biomes. Our study supports the hypothesis directly through our detection of *Pseudomonas* sp. and *Erwinia* sp. that contain specific genes enabling these organisms to facilitate ice-nucleating cloud formation^114^.

However, using eDNA metabarcoding as an ecological monitoring tool comes with some inherent challenges and potential pitfalls^102,115^. Depending on the particular application, eDNA metabarcoding may not provide the ideal data to allow for developing and testing certain hypotheses. For instance, after reviewing the current challenges, limitations, and benefits of using eDNA metabarcoding to study population genetics (e.g., to assess allelic diversity within populations), it is predicted that eDNA monitoring will play an important yet, in some cases, complementary role that will lead to broad-scale inference relating to species population genetics and conservation^116^.

Next logical steps for the further study of the efficacy in using eDNA within throughfall as a biodiversity monitoring tool for terrestrial ecosystems ought to include the continued examination of addressing aforementioned biases and challenges^57,117^, and extending these methods to include the detection eukaryotes as well. Using field methodologies described here, or other standardized methods for sampling throughfall and stemflow^66,67,118^, one could simply modify the sample processing methods and sequencing primers. For example, filtering collected samples to physically sort prokaryotic organisms and using taxa-specific primers would increase the taxonomic resolution of taxonomically-assigned reads within samples^119,120^. The use of 18S ribosomal gene (rDNA) primers to sequence ASVs associated with eukaryotes^121^ is poised to become a widely-used method for detecting arthropods or vertebrates that occur in difficult-to-sample forest canopy habitat. Indeed, recent studies that have sampled eDNA from surfaces within terrestrial ecosystems provide exciting validation of eDNA metabarcoding for detecting insects^38,122^ and vertebrates including amphibians^54,123^, reptiles^56^, and mammals^32^. We also suggest conducting further controlled experiments within the laboratory that would enable us to ‘seed’ simplified vegetated microcosms with known microbial consortia, in order to evaluate various methods of sample collection that might include the spraying of vegetation with sterile de-ionized water, while accounting for effects of DNA degradation similar to previous studies. These steps would be helpful in explicitly modeling and addressing the large bias that we acknowledge is associated with variability introduced by the dynamics of rain-providing weather events themselves. Given the support of the bioprecipitation hypothesis, this phenomenon inherently introduces bias potentially during both sample collection and data processing and analysis (e.g., taxonomic assignment). For these reasons, we envision a field-based experiment that would simulate rain (albeit on a reduced spatial scale), using sterile water, and pumping this simulated rain above the canopy, and collecting samples of throughfall and stemflow to attempt to get a representative and less biased sample of forest-associated communities.

Our demonstration of using a nascent method to measure biodiversity within terrestrial ecosystems additionally holds promise for detecting eukaryotic organisms as well. In a recent controlled study, researchers were able to use eDNA metabarcoding to discriminate between two deer species after sampling recently-browsed twigs containing deer saliva^124^. These results, although from a controlled study, where samples were collected immediately after deer species were in direct contact with the twigs, coupled with our findings warrant conducting future studies aimed at detecting other taxa, including eukaryotes within forest ecosystems. These methods hold much promise for improving our knowledge and ability to measure biodiversity within forest ecosystems, which remains a critical research need given the wide-spread losses in biodiversity due to forest habitat loss and other anthropogenic factors that threaten forest ecosystems globally.

## Conclusion

Despite current potential challenges and potential biases that need to be worked out through future study, our results have given us a promising look into how eDNA metabarcoding can be used to characterize terrestrial forest ecosystem microbial communities, and contribute to a more accurate description of community structure and function. Even if we are not quite at the point where using eDNA metabarcoding will be a viable method to assess population genetic diversity, perhaps through building upon recent advances in our understanding of predicting genotype–phenotype relationships^125,126^, in conjunction with taking a functional-trait approach to modeling generalizable ecosystem dynamics^11^, we will be headed towards a powerful methodology for monitoring global-scale population dynamics among species^39^. We are confident that eDNA metabarcoding will increasingly become a standardized and valuable tool for monitoring global biodiversity, thereby reducing unnecessary costs and impacts from sampling organisms directly, and will find increasing utility for detecting invasive species, detecting cryptic or rare species, and will ultimately help conserve endangered species, and ecological communities associated with imperiled habitats on Earth.

## Supplementary Information

Supplementary Figure.
