# Supplementary material for: Assessing the efficacy of eDNA metabarcoding for measuring microbial biodiversity within forest ecosystems
Source: Sci Rep. 2021 Jan 15;11:1629. doi: 10.1038/s41598-020-80602-9 (PMC7811025; doi:10.1038/s41598-020-80602-9)

SUPPLEMENTARY MATERIALS

***Title:*** Assessing the efficacy of eDNA metabarcoding for measuring microbial biodiversity within forest ecosystems

***Authors:*** Zachary S. Ladin*^a^, Barbra Ferrell^b^, Jacob Dums^c^, Ryan Moore^b^, Delphis F. Levia^d^, W. Gregory Shriver^e^, Vincent D’Amico^f^, Tara L. E. Trammell^a^, João Carlos Setubal^g^, K. Eric Wommack^a^.

***Author Affiliations***: ^a^Department of Plant and Soil Sciences, University of Delaware, 264 Townsend Hall, Newark, Delaware, 19716, USA, ^b^Department of Plant and Soil Sciences, University of Delaware, Delaware Biotechnology Institute, Newark, Delaware, 19716, USA, ^c^ Biotechnology Program, North Carolina State University, Raleigh, NC, 27695, USA, ^d^Department of Entomology and Wildlife Ecology, University of Delaware, 250 Townsend Hall, Newark, Delaware, 19716, USA, ^e^Departments of Geography and Spatial Sciences and Plant and Soil Sciences, University of Delaware, 216C Pearson Hall, Newark, Delaware, 19716, USA, ^f^US Forest Service, Northern Research Station, Newark, DE, USA, ^g^Instituto de Química, University of Sao Paulo, São Paulo, SP 05508-000, Brazil

TABLES

Table S1. List of rainwater samples with corresponding location, type, volume, DNA concentration, and ASV count information.

| Sample_ID | Location | Replicate | Sample_type | Sample_Vol_mL | DNA_Conc_ng_per_uL | ASV_count | ReadCount |
| --- | --- | --- | --- | --- | --- | --- | --- |
| 1 | Control_1 | 1 | ddH_2_O | 30 | < 0.050 | 138 | 1469 |
| 2 | Control_2 | 1 | ddH_2_O | 30 | < 0.050 | 3013 | 58506 |
| 3 | C8_1 | 1 | ddH_2_O | 30 | < 0.050 | 74 | 539 |
| 4 | C8_2 | 1 | ddH_2_O | 39 | < 0.050 | 165 | 674 |
| 5 | E5_1 | 1 | ddH_2_O | 33 | < 0.050 | 62 | 339 |
| 6 | E5_2 | 1 | ddH_2_O | 40 | 0.052 | 973 | 13253 |
| 7 | G7_1 | 1 | ddH_2_O | 37 | 0.055 | 449 | 6628 |
| 8 | G7_2 | 1 | ddH_2_O | 31 | < 0.050 | 182 | 5011 |
| 9 | Control_1 | 1 | Rainwater | 33 | < 0.050 | 57 | 101 |
| 10 | Control_1 | 2 | Rainwater | 32.5 | < 0.050 | 324 | 3352 |
| 11 | Control_2 | 1 | Rainwater | 1 | < 0.050 | 2535 | 36921 |
| 12 | C8_1 | 1 | Throughfall | 9 | 0.55 | 2174 | 21067 |
| 13 | C8_1 | 2 | Throughfall | 30 | 0.559 | 2869 | 67538 |
| 14 | E5_1 | 1 | Throughfall | 7.5 | 0.547 | 2726 | 53787 |
| 15 | E5_1 | 2 | Throughfall | 35 | 2.59 | 3176 | 142228 |
| 16 | E5_2 | 1 | Throughfall | 25 | 1.83 | 2844 | 65544 |
| 17 | E5_2 | 2 | Throughfall | 35 | 2.31 | 2940 | 90493 |
| 18 | G7_1 | 1 | Throughfall | 30 | 0.562 | 1856 | 20210 |
| 19 | G7_1 | 2 | Throughfall | 30 | 0.556 | 255 | 3357 |
| 20 | G7_2 | 1 | Throughfall | 22 | 1.54 | 3017 | 60364 |
| 21 | G7_2 | 2 | Throughfall | 35 | 1.46 | 111 | 1694 |

Table S2. Table of 63 amplicon sequence variants (ASVs) that were uniquely detected within ddH2O samples used as internal standards within our study which were omitted from community analyses.

| **ASV** | **Domain** | **Phylum** | **Class** | **Order** | **Family** | **Genus** | **Species** |
| --- | --- | --- | --- | --- | --- | --- | --- |
| Asv2720 | Bacteria | Acidobacteria | Vicinamibacteria | NA | Vicinamibacteraceae | *Luteitalea* | *pratensis* |
| Asv3260 | Bacteria | Acidobacteria | Blastocatellia | Blastocatellales | Blastocatellaceae | *Aridibacter* | NA |
| Asv787 | Bacteria | Acidobacteria | Blastocatellia | Blastocatellales | Pyrinomonadaceae | *Arenimicrobium* | *luteum* |
| Asv2323 | Bacteria | Actinobacteria | Thermoleophilia | Solirubrobacterales | NA | NA | NA |
| Asv2566 | Bacteria | Actinobacteria | Actinobacteria | Propionibacterineae | Nocardioidaceae | NA | NA |
| Asv2569 | Bacteria | Actinobacteria | Actinobacteria | Micromonosporineae | Micromonosporaceae | *Virgisporangium* | NA |
| Asv3052 | Bacteria | Actinobacteria | NA | NA | NA | NA | NA |
| Asv3264 | Bacteria | Actinobacteria | Actinobacteria | Micrococcales | Microbacteriaceae | NA | NA |
| Asv3492 | Bacteria | Actinobacteria | Rubrobacteridae | Gaiellales | Gaiellaceae | *Gaiella* | *occulta* |
| Asv3495 | Bacteria | Actinobacteria | Actinobacteria | Corynebacterineae | Nocardiaceae | *Williamsia* | *faeni* |
| Asv3751 | Bacteria | Actinobacteria | Actinobacteria | Propionibacterineae | Nocardioidaceae | *Marmoricola* | NA |
| Asv3757 | Bacteria | Actinobacteria | Thermoleophilia | Solirubrobacterales | Solirubrobacteraceae | *Solirubrobacter* | NA |
| Asv4098 | Bacteria | Actinobacteria | Actinobacteria | Frankiales | Frankiaceae | *Jatrophihabitans* | Soli |
| Asv4102 | Bacteria | Actinobacteria | Acidimicrobiia | Acidimicrobiales | Acidimicrobiaceae | NA | NA |
| Asv4106 | Bacteria | Actinobacteria | NA | NA | NA | NA | NA |
| Asv4122 | Bacteria | Actinobacteria | Thermoleophilia | Solirubrobacterales | NA | NA | NA |
| Asv4123 | Bacteria | Actinobacteria | Actinobacteria | Micrococcales | Intrasporangiaceae | *Phycicoccus* | Soli |
| Asv2325 | Bacteria | Bacteroidetes | Chitinophagia | Chitinophagales | Chitinophagaceae | NA | NA |
| Asv239 | Bacteria | Bacteroidetes | NA | NA | NA | NA | NA |
| Asv2444 | Bacteria | Bacteroidetes | Cytophagia | Cytophagales | NA | *Chryseolinea* | *serpens* |
| Asv2575 | Bacteria | Bacteroidetes | Sphingobacteriia | Sphingobacteriales | Sphingobacteriaceae | *Mucilaginibacter* | NA |
| Asv3752 | Bacteria | Bacteroidetes | Chitinophagia | Chitinophagales | Chitinophagaceae | NA | NA |
| Asv4115 | Bacteria | Bacteroidetes | Chitinophagia | Chitinophagales | Chitinophagaceae | *Flavitalea* | *populi* |
| Asv3498 | Bacteria | Firmicutes | Bacilli | Bacillales | Bacillaceae | *Anoxybacillus* | NA |
| Asv3499 | Bacteria | Firmicutes | Bacilli | Lactobacillales | Streptococcaceae | *Streptococcus* | *dysgalactiae* |
| Asv3748 | Bacteria | Fusobacteria | Fusobacteria | Fusobacteriales | Fusobacteriaceae | *Psychrilyobacter* | *atlanticus* |
| Asv2721 | Bacteria | Planctomycetes | Planctomycetes | Planctomycetes | Gemmataceae | *Fimbriiglobus* | *ruber* |
| Asv2889 | Bacteria | Planctomycetes | Planctomycetes | Planctomycetes | Gemmataceae | *Fimbriiglobus* | *ruber* |
| Asv3262 | Bacteria | Planctomycetes | Planctomycetes | Planctomycetes | Isosphaeraceae | NA | NA |
| Asv3764 | Bacteria | Planctomycetes | Phycisphaerae | Tepidisphaerales | Tepidisphaeraceae | *Tepidisphaera* | *mucosa* |
| Asv3768 | Bacteria | Planctomycetes | Planctomycetes | Planctomycetes | Isosphaeraceae | *Singulisphaera* | NA |
| Asv4101 | Bacteria | Planctomycetes | Planctomycetes | Planctomycetes | Gemmataceae | *Fimbriiglobus* | *ruber* |
| Asv4110 | Bacteria | Planctomycetes | Planctomycetes | Planctomycetes | Gemmataceae | *Fimbriiglobus* | *ruber* |
| Asv4120 | Bacteria | Planctomycetes | Planctomycetes | Planctomycetes | Planctomycetaceae | NA | NA |
| Asv4124 | Bacteria | Planctomycetes | Planctomycetes | Planctomycetes | Gemmataceae | *Gemmata* | *obscuriglobus* |
| Asv4129 | Bacteria | Planctomycetes | Phycisphaerae | Tepidisphaerales | Tepidisphaeraceae | *Tepidisphaera* | *mucosa* |
| Asv1403 | Bacteria | Proteobacteria | Deltaproteobacteria | Myxococcales | Cystobacteraceae | *Cystobacter* | *badius* |
| **ASV** | **Domain** | **Phylum** | **Class** | **Order** | **Family** | **Genus** | **Species** |
| Asv1747 | Bacteria | Proteobacteria | Alphaproteobacteria | NA | NA | NA | NA |
| Asv2034 | Bacteria | Proteobacteria | Alphaproteobacteria | Caulobacterales | Caulobacteraceae | NA | NA |
| Asv2207 | Bacteria | Proteobacteria | Deltaproteobacteria | Myxococcales | Polyangiaceae | NA | NA |
| Asv2321 | Bacteria | Proteobacteria | Alphaproteobacteria | Rhizobiales | Bradyrhizobiaceae | *Bradyrhizobium* | *guangdongense* |
| Asv2722 | Bacteria | Proteobacteria | Betaproteobacteria | Burkholderiales | Alcaligenaceae | *Derxia* | *lacustris* |
| Asv3057 | Bacteria | Proteobacteria | Alphaproteobacteria | Rhodospirillales | Acetobacteraceae | NA | NA |
| Asv3256 | Bacteria | Proteobacteria | Gammaproteobacteria | Pseudomonadales | Pseudomonadaceae | *Pseudomonas* | NA |
| Asv3270 | Bacteria | Proteobacteria | Betaproteobacteria | Burkholderiales | Comamonadaceae | NA | NA |
| Asv3493 | Bacteria | Proteobacteria | Gammaproteobacteria | Lysobacterales | Sinobacteraceae | *Povalibacter* | *uvarum* |
| Asv3494 | Bacteria | Proteobacteria | Alphaproteobacteria | Rhodospirillales | Rhodospirillaceae | *Dongia* | *rigui* |
| Asv3496 | Bacteria | Proteobacteria | Gammaproteobacteria | Lysobacterales | NA | NA | NA |
| Asv369 | Bacteria | Proteobacteria | Deltaproteobacteria | Myxococcales | Cystobacteraceae | *Cystobacter* | NA |
| Asv3747 | Bacteria | Proteobacteria | Alphaproteobacteria | Caulobacterales | Caulobacteraceae | *Caulobacter* | *daechungensis* |
| Asv3749 | Bacteria | Proteobacteria | Gammaproteobacteria | NA | NA | NA | NA |
| Asv4097 | Bacteria | Proteobacteria | Betaproteobacteria | Burkholderiales | NA | NA | NA |
| Asv4100 | Bacteria | Proteobacteria | Alphaproteobacteria | Sphingomonadales | Sphingomonadaceae | *Sphingomonas* | *lutea* |
| Asv4113 | Bacteria | Proteobacteria | Betaproteobacteria | Burkholderiales | Comamonadaceae | *Polaromonas* | *aquatica* |
| Asv4119 | Bacteria | Proteobacteria | Alphaproteobacteria | Rhodospirillales | Rhodospirillaceae | NA | NA |
| Asv4128 | Bacteria | Proteobacteria | Alphaproteobacteria | Sphingomonadales | Sphingomonadaceae | *Sphingomonas* | *oligophenolica* |
| Asv4134 | Bacteria | Proteobacteria | Betaproteobacteria | Burkholderiales | Comamonadaceae | *Limnohabitans* | *parvus* |
| Asv4136 | Bacteria | Proteobacteria | Betaproteobacteria | Burkholderiales | Comamonadaceae | NA | NA |
| Asv4137 | Bacteria | Proteobacteria | Betaproteobacteria | Burkholderiales | NA | NA | NA |
| Asv2882 | Bacteria | Verrucomicrobia | Verrucomicrobiae | Verrucomicrobiales | Verrucomicrobiaceae | NA | NA |
| Asv3753 | Bacteria | Verrucomicrobia | Spartobacteria | NA | NA | *Terrimicrobium* | *sacchariphilum* |
| Asv4107 | Bacteria | Verrucomicrobia | Verrucomicrobiae | Verrucomicrobiales | NA | NA | NA |

Figure S1. Image showing paired replicate rainwater collection methods using funnels within a forest sampling location in Newark, Delaware, USA.


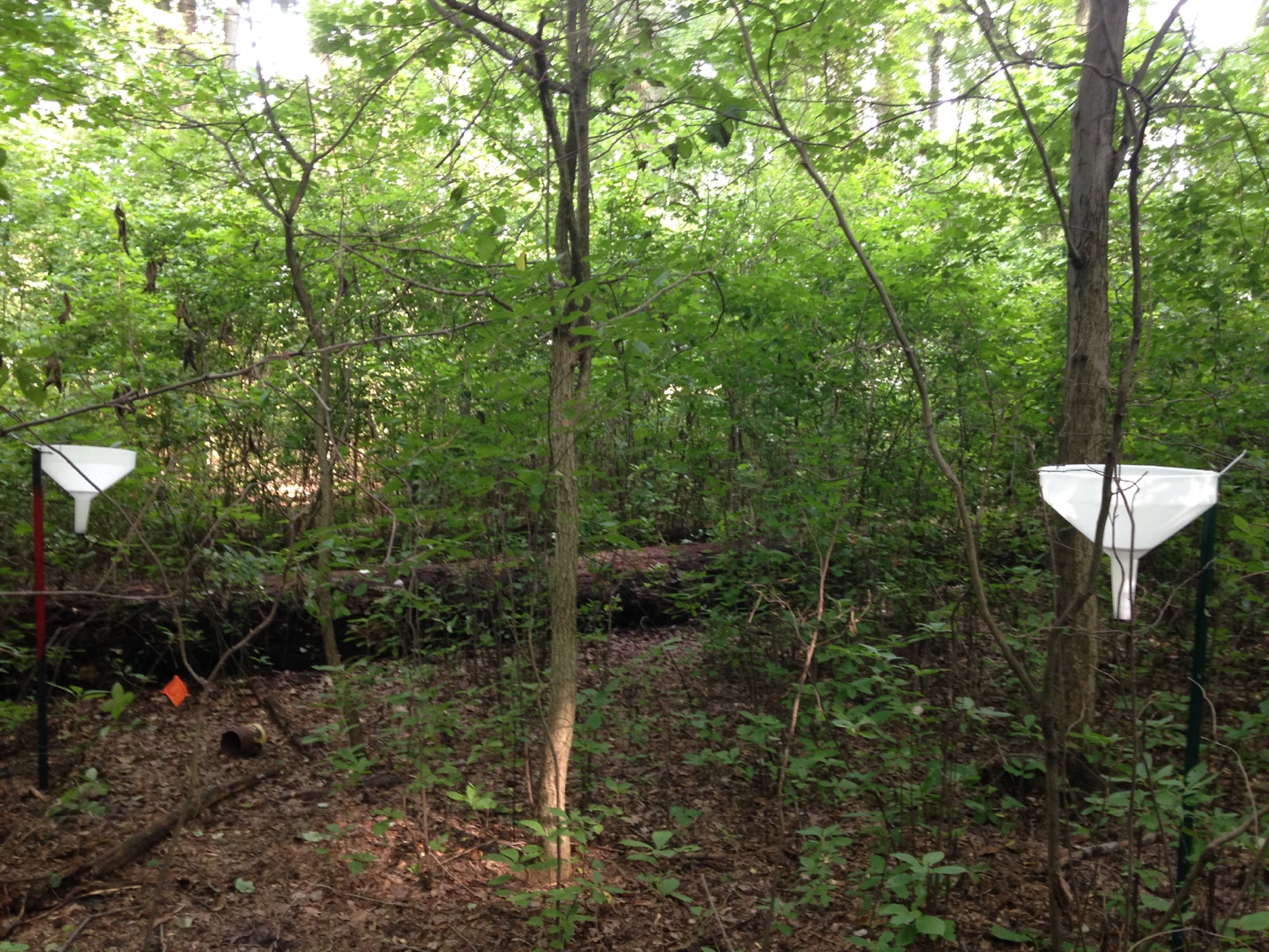


Figure S2. Rarefaction curve showing total reads and number of amplicon sequence variants (ASVs) per sampling location (*N* = 14) that was used to determine the threshold for inclusion of samples having > 2000 (solid black vertical line) reads within analyses.


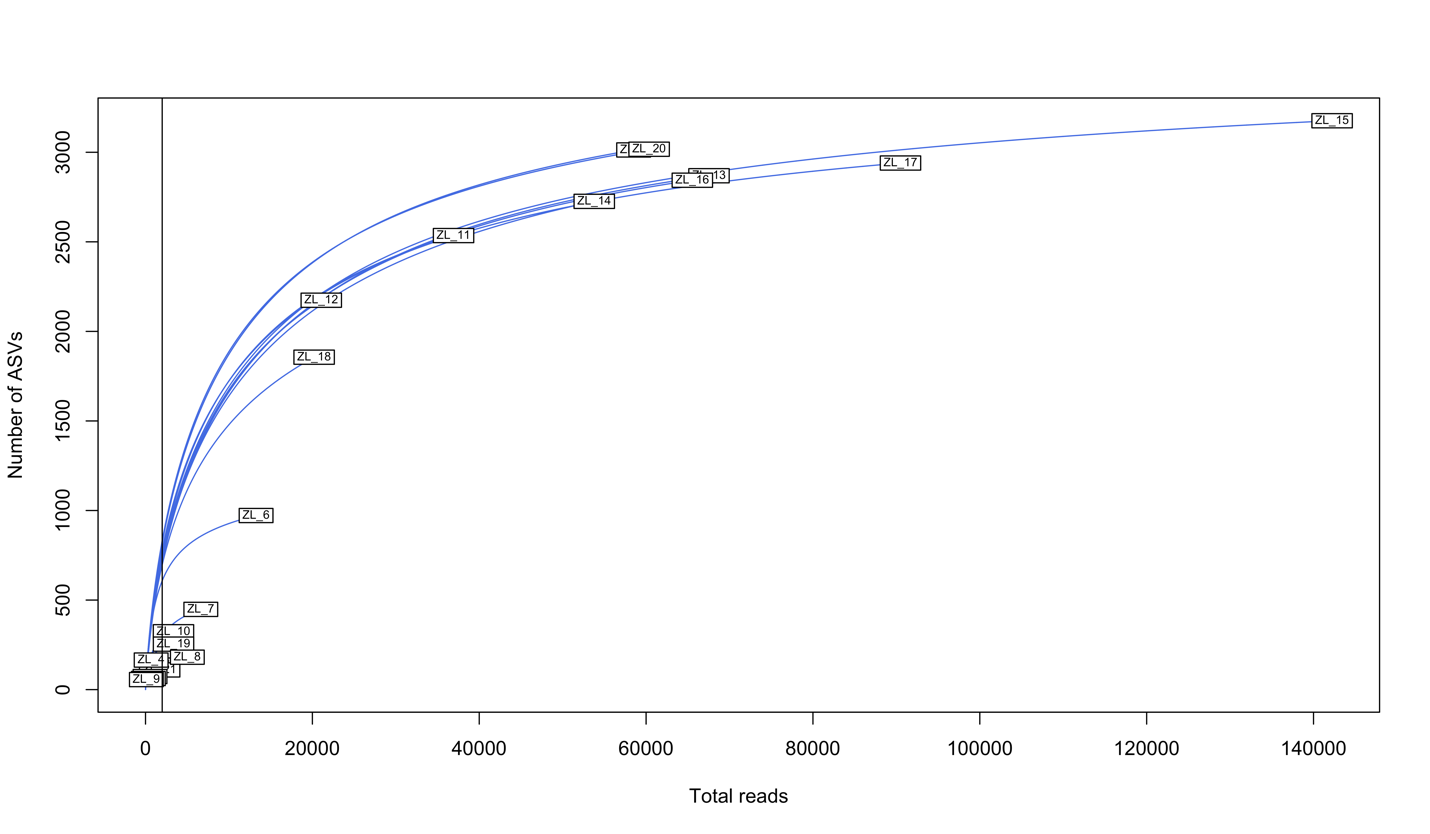


Figure S3. Number of total reads of amplicon sequence variants (ASVs) per sampling location (*N* = 14). ASVs are shown from ddH_2_O (gray) and rainwater and throughfall (blue) samples. The dotted line demarks a threshold of 2,000 total reads for samples, below which samples were excluded in diversity analyses.


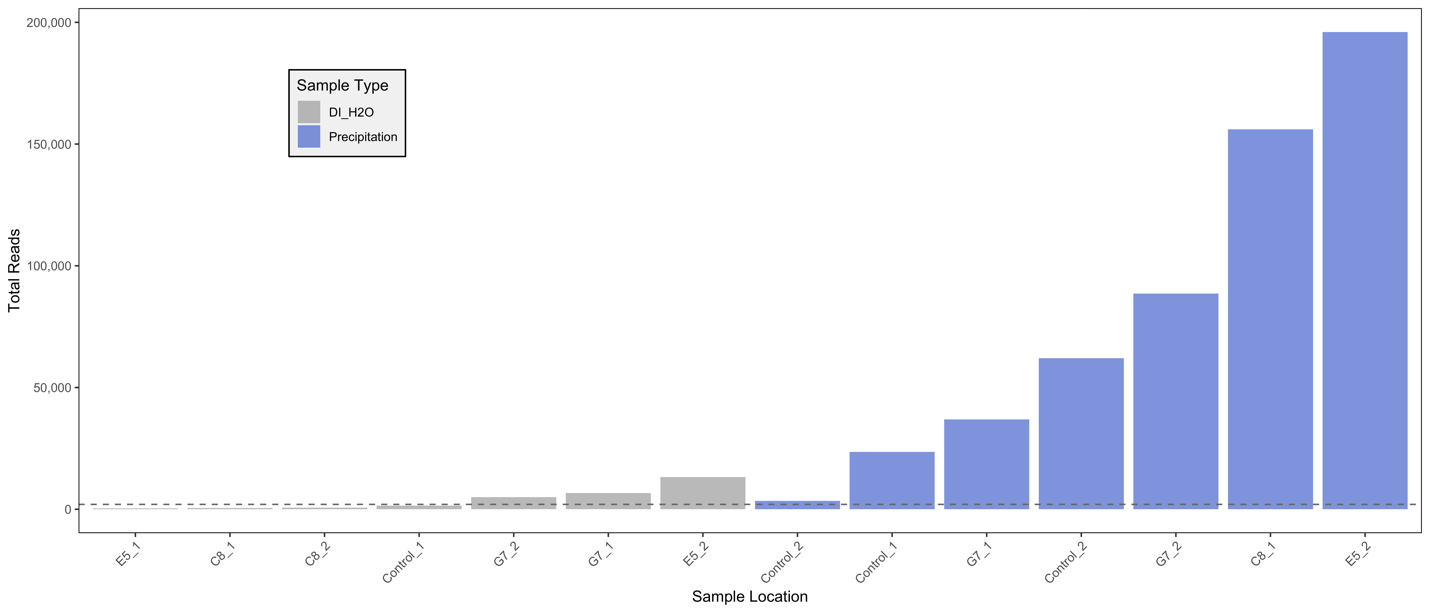

Supplement: Supplementary file 1 — Supplementary Figure. [file 41598_2020_80602_MOESM1_ESM.docx]
